# The complete mitochondrial genome of enigmatic mycoparasitic fungus *Squamanita imbachii*

**DOI:** 10.1080/23802359.2024.2356131

**Published:** 2024-06-10

**Authors:** Jian-Wei Liu, Thatsanee Luangharn, Fu-Qiang Yu, Chitrabhanu S. Bhunjun

**Affiliations:** a Germplasm Bank of Wild Species & Yunnan Key Laboratory for Fungal Diversity and Green Development, Kunming Institute of Botany, Chinese Academy of Sciences, Kunming, China; b Center of Excellence in Fungal Research, Mae Fah Luang University, Mueang Chiang Rai, Thailand; c School of Science, Mae Fah Luang University, Mueang Chiang Rai, Thailand

**Keywords:** Agaricales, fungicolous fungi, phylogeny, Squamanitaceae

## Abstract

The complete mitochondrial genome of *Squamanita imbachii* I. Saar, is unveiled in this research for the first time. It covers 76,643 base pairs (bp) and exhibits a guanine-cytosine (GC) content of 23%. The genome includes 14 conserved protein-coding genes, 1 DNA polymerase gene, 2 ribosomal RNA gene (RNS and RNL), 25 transfer RNA (tRNA) genes and 18 open reading frames (ORFs). Phylogenetic analysis, utilizing a mitochondrial gene dataset from 15 taxa across seven families within the Agaricales order, was conducted employing the maximum-likelihood (ML) approach. This analysis identified a close phylogenetic relationship between *S. imbachii* and *Floccularia luteovirens* (Alb. & Schwein.) Pouzar 1957, positioning both within the Squamanitaceae family.

## Introduction

*Squamanita* is a genus in the family Squamanitaceae (Vizzini et al. 2019; Kalichman et al. 2020; Liu et al. 2021), which may represent one of the most unique basidiomycetes fungi in the world as nearly all the species are parasitic on other living fungi. Saar et al. (2022) split *Squamanita* into two monophyletic groups, viz. *Squamanita* and *Dissoderma* (A.H. Sm. & Singer) Singer 1973. Fourteen and nine species are accepted in *Squamanita* and *Dissoderma* worldwide, respectively (Species Fungorum 2024).

**Figure 1.**
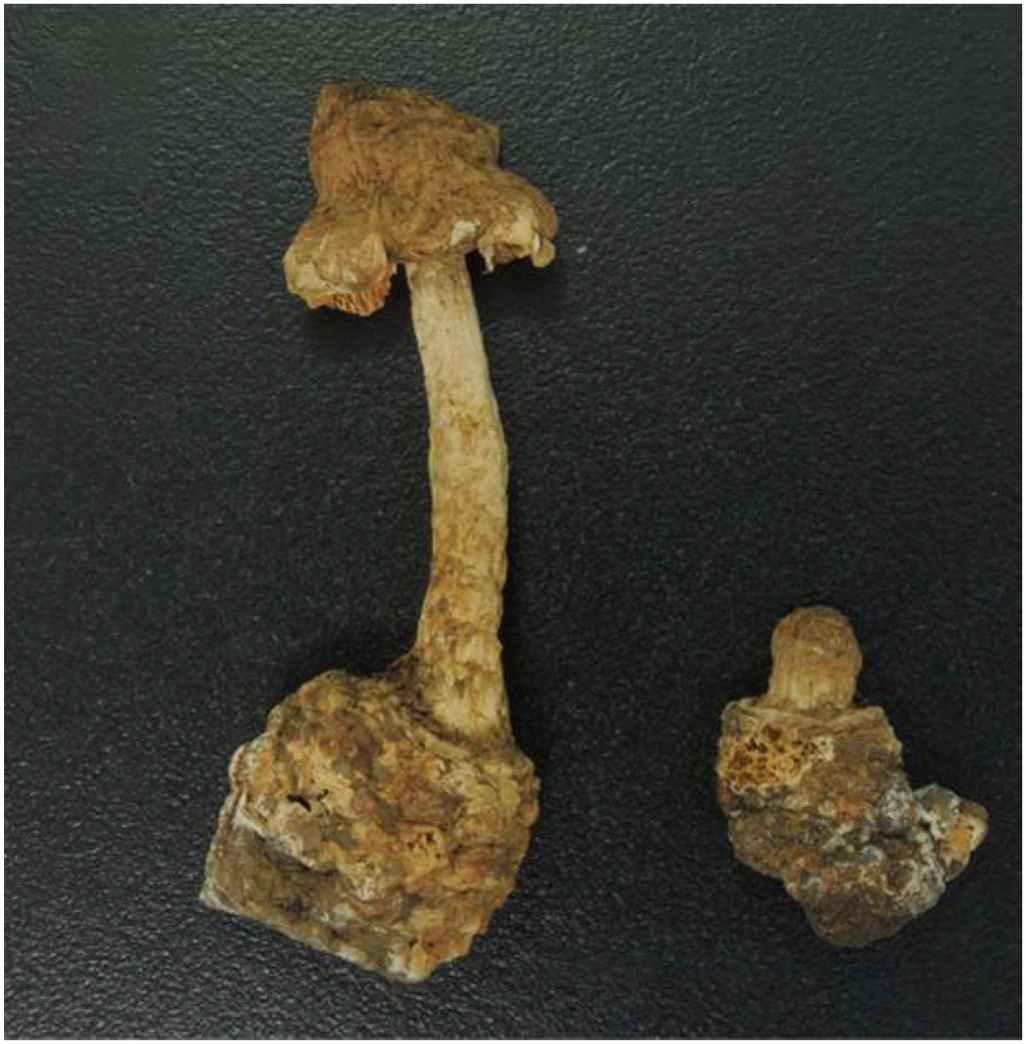
Image of *Squamanita imbachii* (HKAS107325A) sequenced in this work Liu et al. (2021).

*Squamanita imbachii* has been documented in Europe and North America (Liu et al. 2021; Saar et al. 2022). Liu et al. (2021) sequenced a specimen (HKAS107325A) collected from Liguria, Italy, resulting in the generation of four genes (ITS: MW258856; LSU: MW258908; TEF1-α: MW324508; 18S: MW258935 & MW258886) and noted that this species acts as a parasite on *Amanita excelsa* species complex (Fr.) Bertill. 1866. Saar et al. (2022) confirmed the identity of HKAS107325A as *S. imbachii*. In this study, we used this dried specimen archived in the Herbarium of Cryptogams, Kunming Institute of Botany, Chinese Academy of Sciences (http://www.kib.ac.cn/) (Figure 1).

## Materials and methods

### Genomic DNA extraction and sequencing

The CTAB method (Doyle and Doyle 1987) was utilized to extract the entire genomic DNA from the desiccated *S. imbachii* specimen. Standard protocols were followed for whole-genome sequencing on an Illumina sequencing platform (HiSeq PE150). Paired-end libraries with 150 bp were created, resulting in approximately 6 GB of raw data.

### Assembly and annotation

The assembly process was carried out employing GetOrganelle v. 1.7.5 with default settings (Jin et al. 2020). Initial annotation was automated using the MFannot tool (Beck and Lang 2010), relying on genetic code 4 (Mold, Protozoan, and Coelenterate Mitochondrial genetic code). Verification of rRNA and tRNA genes was performed using RNAweasel (Beck and Lang 2010) and tRNAscan-SE 2.0 (Lowe and Chan 2016), respectively. To ensure accurate annotation, the intron and exon boundaries of Protein-Coding Genes (PCGs) were individually validated by aligning gene sequences with homologous alleles sequences (obtained *via* Extract Regions in Geneious Prime) from previously annotated mitogenomes of Squamanitaceae using the MAFFT program in Geneious Prime 2020.0.3 (BioMatters, Ltd., Auckland, New Zealand). OGDraw v. 1.2 (Lohse et al. 2013) was employed to generate a physical circular map of *S. imbachii*. Sequencing depth and coverage was calculated following the procedure outlined in Ni et al. (2023).

### Phylogenetic analysis

We constructed a phylogenetic tree for 15 Agaricineae species to validate the phylogenetic status of *S. imbachii. Amanita phalloides* (Vaill. ex Fr.) Link 1833 and *A. basii* Guzmán & Ram. Guill. 2001 from Pluteineae were selected as the outgroup. Fifteen common conserved PCGs sequences were extracted, aligned, and concatenated using Geneious Prime 2020.0.3. Partitioned substitution model (ATP6: LG; ATP8: MTZOA; ATP9: HIVB; COB: MTZOA; COX1: CPREV; COX2: MTZOA; COX3: MTZOA; NAD1: MTZOA; NAD2: LG; NAD3: MTZOA; NAD4L: LG; NAD4: LG; NAD5: LG; NAD6: CPREV; RPS3: CPREV) evaluation and phylogenetic tree construction for maximum likelihood (ML) analysis were performed using RAxML.8.2.12 (Stamatakis 2006).

## Results

**Figure 2.**
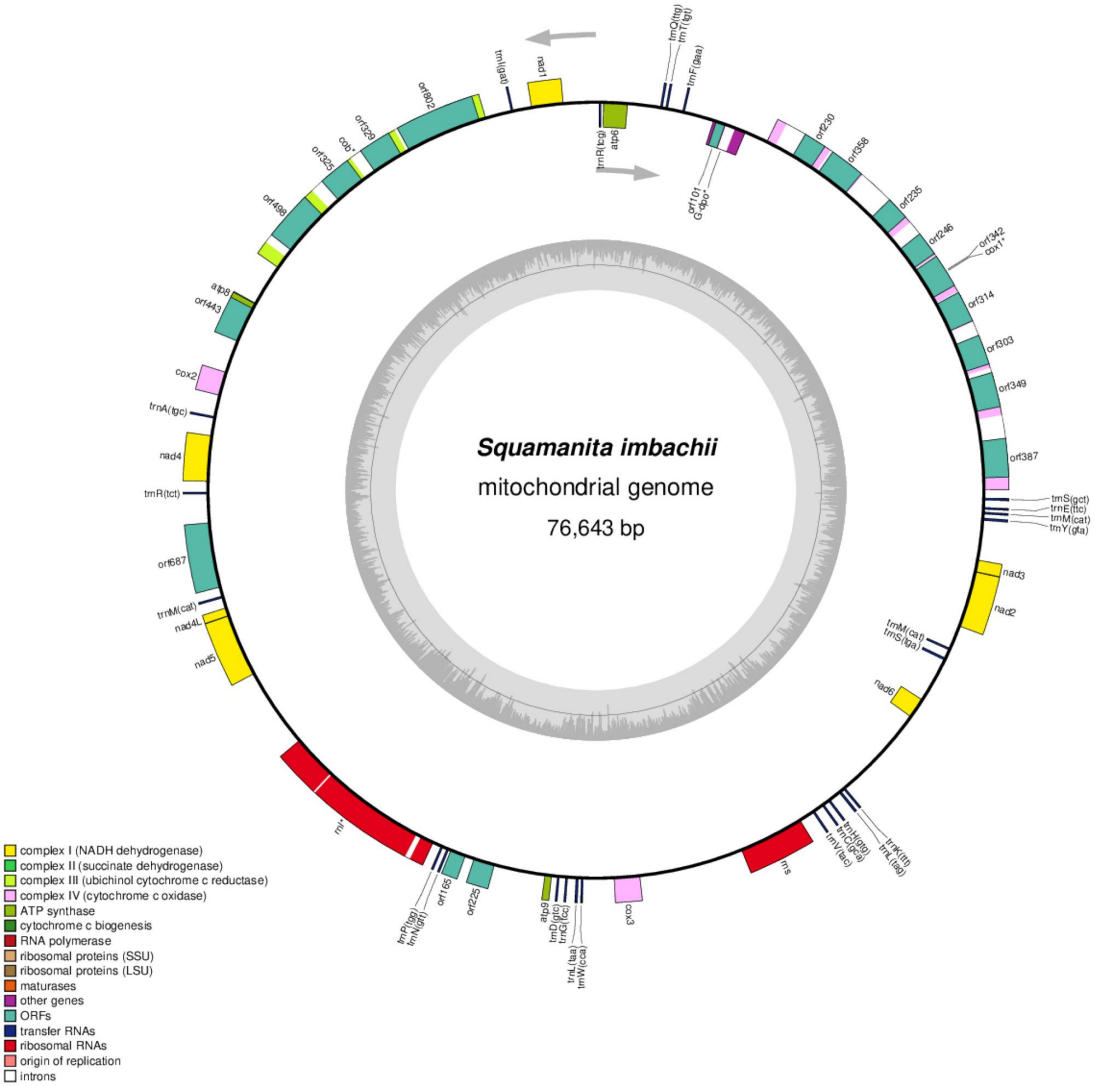
Circular map of the mitochondrial genomes of *S. imbachii*. Genes are represented by different colored blocks. Colored blocks outside each ring indicate that the genes are on the direct strand, while colored blocks within the ring indicate that the genes are located on the reverse strand. The inner grayscale bar graph shows the GC content of the mitochondrial sequences. The circle inside the GC content graph marks the 50% threshold. Label intron-containing genes with *.

The complete mitochondrial genome sequence of *S. imbachii* has been deposited in GenBank (accession no. PP417754; Figure 2). The circular genome (76,643 bp) comprised 14 conserved protein-coding genes (PCGs), 2 rRNA gene (RNS and RNL), 25 transfer RNA (tRNA) genes, 1 DNApolymerase gene (likely a pseudo-gene, introduced by plasmid integration) and 18 open reading frames (ORFs). Among these, 13 ORFs originate from the COX1 and COB genes, which are intron-encoded. Within the COX1 gene, eight out of nine ORFs belong to the LAGLIDADG type, while one belongs to the GIY type. Among the introns the involved encoded ORFs, seven belong to group I, one belongs to group II, and one remains unassigned. Regarding the COB gene, two out of four ORFs are classified as LAGLIDADG type, one as GIY type, and three out of four introns involved encoded ORFs belong to group I, while one belongs to group II. Becides, RNL gene also include two introns belong to group I and group II, respectively. The 14 conserved PCGs respectively encoded the seven ubiquinone reductase subunits of NADH (NAD1, NAD2, NAD3, NAD4, NAD4L, NAD5, and NAD6), three cytochrome oxidase subunits (COX1, COX2, and COX3), three ATP synthase subunits (ATP6, ATP8, and ATP9), and apocytochrome b (COB). The 25 tRNA genes (tRNA^Ala(TGC)^, tRNA^Cys(GCA)^, tRNA^Asp(GTC)^, tRNA^Glu(TTC)^, tRNA^Phe(GAA)^, tRNA^Gly(TCC)^, tRNA^His(GTG)^, tRNA^Ile(GAT)^, tRNA^Lys(TTT)^, tRNA^Leu(TAA)^, tRNA^Leu(TAG)^, tRNA^Met(CAT)^, tRNA^Asn(GTT)^, tRNA^Pro(TGG)^, tRNA^Gln(TTG)^, tRNA^Arg(TCG)^, tRNA^Arg(TCT)^, tRNA^Ser(GCT)^, tRNA^Ser(TGA)^, tRNA^Thr(TGT)^, tRNA^Val(TAC)^, tRNA^Trp(CCA)^, tRNA^Tyr(GTA)^ ranged from 71 to 85 bp, and covered all 20 standard amino acids. The maximum, minimum and average sequencing depth was 8027, 6110, and 7914.37, respectively (Figure S1). The overall base composition is as follows: 37.81% A, 38.84% T, 11.30% C, and 12.05% G, with a GC content of 23%.

**Figure 3.**
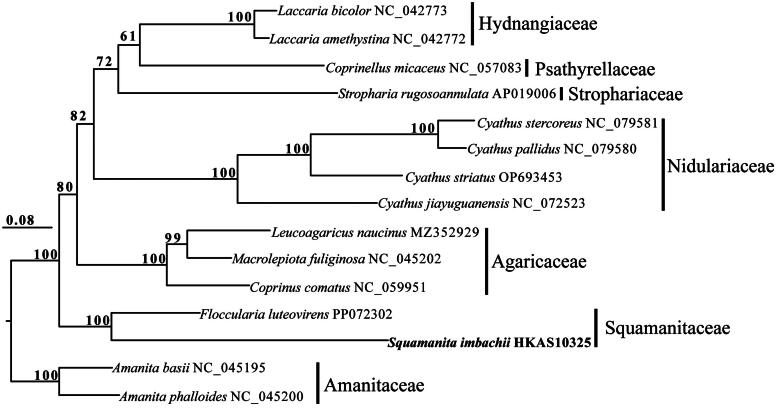
Phylogenetic relationships among 15 species based on concatenated mitochondrial protein-coding genes (PCGs) using partitioned models. The 15 PCGs included subunits of the respiratory chain complexes (COB, COX1, COX2, and COX3), ATPase subunits (ATP6, ATP8, and ATP9), NADH: quinone reductase subunits (NAD1, NAD2, NAD3, NAD4, NAD4L, NAD5, and NAD6), and ribosomal protein (RPS3). Support values are shown at the corresponding nodes. Accession numbers of mitochondrial sequences used in the phylogenetic analysis are listed after the species names.

*Squamanita imbachii* and *F. luteovirens* exhibited a close relationship in the phylogenetic tree and both of them belonged to Squamanitaceae (Figure 3).

## Discussion and conclusion

In this study, we conducted sequencing and characterization of the first mitochondrial genome from the rare genus *Squamanita*. Phylogenetic analysis revealed that *S. imbachii* and *F. luteovirens* are affiliated with Squamanitaceae based on mitochondrial protein-coding gene, a finding consistent with Liu et al. (2021) that was based on multiple nuclear genes. Notably, our current phylogenetic tree (Figure 3) reveals that the family Squamanitaceae does not cluster with Nidulariaceae. Given the maternally inherited nature of the mitochondrial genome, evolutionary histories infering based on mitochondrial genomes often differ from those based on biparentally inherited nuclear genomes. Further sequencing and the generation of additional mitochondrial genomes from a broader range of representative taxa within Suborder Pluteineae would enhance our understanding of the evolutionary relationships among these remarkable mycoparasitic basidiomycetes fungi.

## Data Availability

The genome sequence data that support the findings of this study are openly available in GenBank of NCBI at https://dataview.ncbi.nlm.nih.gov/under the accession no. PP417754. The associated BioProject, SRA, and Bio-Sample numbers are PRJNA811355, SRP361919, and SAMN40372582, respectively.

## References

[CIT0001] Beck N, Lang B. 2010. MFannot, organelle genome annotation Websever. Montréal QC: Université de Montréal.

[CIT0002] Doyle JJ, Doyle JL. 1987. A rapid DNA isolation procedure for small quantities of fresh leaf tissue. Phytochem Bull. 19:11–15.

[CIT0003] Jin JJ, Yu WB, Yang JB, Song Y, dePamphilis CW, Yi TS, Li DZ. 2020. GetOrganelle: a fast and versatile toolkit for accurate de novo assembly of organelle genomes. Genome Biol. 21(1):241. doi:10.1186/s13059-020-02154-5. 32912315 PMC7488116

[CIT0004] Kalichman J, Kirk PM, Matheny PB. 2020. A compendium of generic names of agarics and Agaricales. Taxon. 69(3):425–447. doi:10.1002/tax.12240.

[CIT0005] Liu JW, Ge ZW, Horak E, Vizzini A, Halling RE, Pan CL, Yang ZL. 2021. Squamanitaceae and three new species of *Squamanita* parasitic on *Amanita* basidiomes. IMA Fungus. 12(1):4. doi:10.1186/s43008-021-00057-z. 33658081 PMC7927255

[CIT0006] Lohse M, Drechsel O, Kahlau S, Bock R. 2013. OrganellarGenomeDRAW–a suite oftools for generating physical maps of plastid and mitochondrial genomesand visualizing expression data sets. Nucleic Acids Res. 41(Web Server issue):W575–W581. doi:10.1093/nar/gkt289. 23609545 PMC3692101

[CIT0007] Lowe TM, Chan PP. 2016. tRNAscan-SE On-line: integrating search and context for analysis of transfer rna genes. Nucleic Acids Res. 44(W1):W54–W57. doi:10.1093/nar/gkw413. 27174935 PMC4987944

[CIT0008] Ni Y, Li J, Zhang C, Liu C. 2023. Generating sequencing depth and coverage map for organelle genomes. protocols.io. doi:10.17504/protocols.io.4r3l27jkxg1y/v1.

[CIT0009] Saar I, Thorn RG, Nagasawa E, Henkel TW, Cooper JA. 2022. A phylogenetic overview of *Squamanita*, with descriptions of nine new species and four new combinations. Mycologia. 114(4):769–797. doi:10.1080/00275514.2022.2059639. 35695889

[CIT0010] Species Fungorum. 2024. http://www.speciesfungorum.org/.

[CIT0011] Stamatakis A. 2006. RAxML-VI-HPC: maximum likelihood-based phylogenetic analyses with thousands of taxa and mixed models. Bioinformatics. 22(21):2688–2690. doi:10.1093/bioinformatics/btl446. 16928733

[CIT0012] Vizzini A, Consiglio G, Marchetti M. 2019. Mythicomycetaceae fam. nov. (Agaricineae, Agaricales) for accommodating the genera *Mythicomyces* and *Stagnicola*, and *Simocybe* parvispora reconsidered. Fungal Syst Evol. 1(1):41–56. doi:10.3114/fuse.2019.03.05. 32467883 PMC7235982

